# Hybrid Analog Computer for Modeling Nonlinear Dynamical Systems: The Complete Cookbook

**DOI:** 10.3390/s23073599

**Published:** 2023-03-30

**Authors:** Miroslav Rujzl, Ladislav Polak, Jiri Petrzela

**Affiliations:** Department of Radio Electronics, Faculty of Electrical Engineering and Communication, Brno University of Technology, Technicka 3082/12, 616 00 Brno, Czech Republic; 196809@vut.cz (M.R.); petrzela@vutbr.cz (J.P.)

**Keywords:** analog computer, circuit synthesis, chaotic system, strange attractor, transfer function

## Abstract

This paper describes a design process for a universal development kit based on an analog computer concept that can model the dynamics of an arbitrarily complex dynamical system up to the fourth order. The constructed development kit contains digital blocks and associated analog-to-digital and digital-to-analog converters (ADCs and DAC), such that multiple-segmented piecewise-linear input–output characteristics can be used for the synthesis of the prescribed mathematical model. Polynomial input–output curves can be implemented easily by four-quadrant analog multipliers. The proposed kit was verified through several experimental scenarios, starting with simple sinusoidal oscillators and ending with generators of continuous-time robust chaotic attractors. The description of each individual part of the development kit is accompanied by links to technical documentation, allowing skilled readers in the construction of electronic systems to replicate the proposed functional example. For this purpose, the electrical scheme of the hybrid analog computer and all important source codes are available online.

## 1. Introduction

The construction of chaotic oscillators has been a favorite topic of analog design engineers and enthusiasts for many decades. The unique properties of continuous-time chaotic signals strongly motivate researchers to develop generators of chaos that are robust with respect to variations in fundamental circuit parameters and non-ideal intrinsic properties of the used active elements. The chaotic oscillators discussed in this paper can be understood as lumped electronic systems where output voltages or currents represent a steady state that is extremely sensitive to tiny changes in initial conditions. These systems exhibit a continuous broad-band frequency spectrum and produce dense strange attractors with a prescribed geometrical shape. These ω-limit sets need to be long-term stable and repeatable, avoiding misinterpretation with chaotic transients.

Based on the complete knowledge of the mathematical model, which involves both ordinary differential equations and numerical values of internal parameters are known, many systematic approaches can result in the circuitry implementation of a chaotic oscillator containing commercially available components only. This paper focuses on one straightforward method that has been known for decades: the analog computer concept. The authors’ main aim is to provide a detailed cookbook for constructing a maximally universal development kit based on the integrator block schematic of a general class of fourth-order dynamical systems. This universality includes arbitrarily shaped polynomial output-input functions up to the fourth order, a nonlinear transfer function with digitally controlled slopes of the piecewise-linear segments, as well as the number and positions of breakpoints, variable complexity of initial state equations (including the possibility to model cyclically symmetrical vector fields and fractional-order nonlinear circuits), and the choice of initial conditions (both positive and negative values) that can be imposed into the circuit. Interested readers will be directed to internet storage sites where all technical documentation can be found, including layouts of individual printed circuit boards, lists of passive and active components, design rules, etc.

This paper is organized as follows: The following section guides readers through a carefully selected collection of papers published on the topic of modeling deterministic nonlinear dynamics via electronic circuits, with particular emphasis on the analog computer concept. The third section discusses the fundamental composition of the development kit, including descriptions of the analog and digital parts, as well as the interfaces between individual functional blocks. The fourth section deals with the construction of the printed circuit board, providing tables with component lists and discussions of design requirements. The fifth section is full of demonstrations, beginning with mathematical models and ending with captured oscilloscope screenshots. This section compares theory and practice, showing excellent agreement between the two. Finally, a few concluding remarks are presented, which mainly focus on additional possible improvements of the proposed functional example.

## 2. Brief Overview of the Systematic Design of Lumped Chaotic Oscillators

One of the earliest lumped chaotic systems was named after its inventor, Prof. L. O. Chua, and was first realized in the early 1980s. This system is still considered one of the simplest circuitry realizations in terms of total component count. It consists of a third-order passive ladder immittance and a five-segment odd-symmetric piecewise linear (PWL) resistor [1,2,3]. After its discovery, Chua’s oscillator underwent many modifications motivated by different requirements, such as removing the real inductor in parallel resonant circuits [4] or enabling high-frequency operations [5]. Despite its age-long birth date, this circuit remains a favorite educational example for demonstrating the birth and death of chaos. It is also a source of chaotic signals with various levels of entropy for practical applications, including modulation and secure communication techniques. It is a tool used for the graphical explanation of chaos evolution and is a subject of advanced numerical analysis in general [6].

The parallel connection of a higher-order two-terminal immittance and a nonlinear resistor is used in other implementations of lumped chaotic systems. For example, reference [7] shows that a passive ladder structure composed of resistors and capacitors can produce chaotic waveforms if terminated by a three-segment PWL active resistor with specific slopes. The main drawbacks of this concept, where the linear part of the vector field is realized by a passive RC two-terminal network, are the lack of universality and transparency. The first disadvantage arises from the passivity of the two-terminal immittance. For given numerical values of parameters of the mathematical model (eigenvalues associated with individual segments of the used PWL function), the two-terminal immittance can contain one or several basic negative elements [8] or second-order accumulation elements [9,10]. The second disadvantage is related to the relationships between the parameters of the original mathematical model and the values of circuit elements because these can be too complicated to count by heart. A generalization of the approach discussed above toward arbitrary-order dimensional dynamical systems is provided in [11]. Both of the drawbacks mentioned above can be removed by adopting a design method based on the integrator block schematics of the dynamical system. It is worth noting that only deterministic systems will be considered in the upcoming sections of this paper.

An analog computer can be thought of as an electronic circuit that directly solves a set of first-order ordinary differential equations. Differentiation is typically achieved using a lossless integrator on the right-hand side of the differential equations; $\dot{x}=f\left(x\right)$ are typically implemented in analog computers using differential amplifiers, inverting ’summators,’ and two ports with the prescribed input–output characteristics. In most cases, individual state variables are voltages, which are easily accessible and measurable at the outputs of integrators [12,13,14]. Although analog computers tend to have a higher number of active elements, these properties can sometimes be reduced by using linear transformations of the coordinates. In current-mode analog computers, simple circuit nodes represent non-weighted summation blocks for currents, making them potentially simpler than their voltage-mode counterparts [15]. Of course, purely for practical reasons, the commercial availability of active elements is the decisive factor for the operational regime of an analog computer. If simulation results are sufficient to verify circuit design, a wide range of hypothetical active elements can be considered, as discussed in [16]. The internal structure of a versatile analog building block fabricated using 350 nm CMOS technology and optimized for the synthesis of complex nonlinear dynamical systems is described in [17]. The authors note that trans-conductance mode active elements are particularly beneficial for the synthesis of chaotic and hyperchaotic electronic systems.

Let’s start with the simplest examples of integrator-based chaotic oscillators. Reference [18] demonstrates several circuit realizations of the so-called jerk functions. In these cases, the second state variable is the derivative of the first one and the third state variable is the time derivative of the second one. Thus, the state variables can be interpreted as position, velocity, and acceleration, making the mathematical model a member of Newtonian mechanics. Starting with mathematical models in the form of a single third-order ordinary differential equation with an absolute value or signum nonlinearity, the final autonomous circuit contains a cascade connection of three ideal inverting integrators supplemented by semiconductor diodes or comparators. Both simulation and experimental results are provided, showing excellent agreement between theory and practice. The jerk dynamics with the absolute value nonlinear function represents the original mathematical model for a chaotic oscillator described in [19]. Phase portraits and bifurcation diagrams calculated for a change in a single resistor are provided, although only the simulation results associated with the final chaotic oscillator are presented. The same design approach associated with the linear part of the vector field, but with more circuit realizations of various nonlinear transfer functions, is offered to readers in [20]. In [21], the authors describe the algebraically simplest jerk function capable of exhibiting robust chaos. The final oscillator contains four standard operational amplifiers, which are available as a single integrated circuit, and one diode. The authors also provide a photo of an analog computer reconfigurable kit, although it is much less universal than the development kit presented in this paper. In [22], the authors substitute two active integrators with passive RC or RLC low-pass filters. Such circuits can be considered a bridge between the two types of realizations mentioned above. A simple jump function is implemented by an operational amplifier-based comparator as the only nonlinearity.

The pure concept of an analog computer can be found in many papers dealing with the design of chaotic systems. For example, a step-by-step description of a chaotic oscillator based on a given set of ordinary differential equations is provided in [23]. There, the authors were able to make a significant discovery: a robust chaotic solution associated with a system having one stable equilibrium. Nine algebraic terms are practically implemented using two analog multipliers and six voltage feedback operational amplifiers. In the work [24], the authors present new chaotic dynamical systems with surfaces of equilibria and their demonstration circuitry realizations. As stated in the paper, real measurements can be considered an alternative to numerical simulations, which are always subject to rounding and approximation errors and require a long time. A similar circuit concept, only with standard operational amplifiers and four-quadrant multipliers, is utilized for experimental verification of systems with only quadratic and cubic terms in the [25]. In the design of a chaotic dynamical system with multiple chaotic attractors presented in [26], four AD633 analog multipliers and the same number of operational amplifiers are the active elements employed. It is demonstrated that each strange attractor revealed during numerical analysis can also be observed in the experimental measurement of the practical circuit. Thus, imposing a set of initial conditions into the proper nodes of the chaotic oscillator is possible and seems to be an easy task. The difficult, but eventually successful circuitry realization of a chaotic system with circular and square equilibrium is the subject of reference [27]. The basin of attraction leading to the desired chaotic attractor is rather narrow and located far away from the origin of the state space. Nevertheless, the authors were able to verify the prescribed chaotic attractor over a long time (because of the chosen short time constant of the utilized integrators) in this case as well. Of course, chaotic dynamical systems having other geometrical shapes of degenerated equilibria can also be modeled using the analog computer approach. For example, in [28], the authors present a chaotic system with a rounded square equilibrium, and both numerical analysis and true experimental measurement show very good agreement. The circuit realization comprises only an inverting summing integrator and analog multipliers AD633, without differential and summing amplifiers. In [29], the authors present twelve new chaotic dynamical systems where a single internal parameter can be used to study the interactions between the generated chaotic attractor and the multiple-dimensional equilibrium structure, such as planes, curved planes, rings, and spheres. For experiments, two chaotic systems are chosen as examples and realized as electronic circuits using cheap and off-the-shelf operational amplifiers LM741. The required quadratic terms are implemented using analog multipliers connected as squarers, with nine and seven such active elements needed for the first and second oscillators, respectively. Chaotic circuits designed using the analog computer approach, especially those employing operational transconductance amplifiers only, can serve as a pattern for full on-chip implementations using CMOS technology [30]. Advantageously, these structures contain no resistors, i.e., the final oscillators occupy very small chip areas.

In cases where the nonlinearity has a very complicated shape, its realization using discrete analog components can be difficult, and even impossible. For example, in [31], the authors focus on the circuit design of a system that generates a multi-wing butterfly attractor. The corresponding mathematical model contains one scalar nonlinear function in the form of a sum of signum jumps with different breakpoints. Since each jump function must be implemented using a comparator, a very complex final circuit can be expected. This assumption is proven correct since the corresponding oscillator contains more than fifty operational amplifiers. The sine function can be approximated by a finite power series containing only even powers of the input variable. Similarly, the cosine function approximation near the origin contains odd powers of the input variable. The utilization of the cosine function in a non-autonomous chaotic oscillator (driven by a single-tone sinusoidal signal) based on the analog computer concept is suggested in [32], although real measurement results are not provided. Both trigonometric functions mentioned above can be used in the so-called labyrinth chaos generator [33]. However, low system dissipation leads to a large state space volume occupied by the desired attractor. Since achieving a wide dynamical range of active devices can be problematic, a digital approach is suggested in [34]. Because the output of digital blocks is not continuously valued smooth function, the resulting mathematical model is piecewise constant rather than PWL. Nevertheless, both simulation and experimental results remain in good agreement, proving this concept of digitalization of nonlinear feedback function.

An extension of the integrator block schematic for arbitrary-dimensional mathematical models is not a problem, making it a good choice for hyperchaotic systems [35]. The analog computer idea can be applied not only to make a circuit equivalent to a given mathematical model but also for accurate dynamical modeling of existing lumped electronic networks of any kind [36,37], including non-electronic systems. However, since each electronic system contains many active elements, their parasitic properties can seriously affect the global dynamics. Thus, to remove unwanted disturbances at higher frequencies, numerical values of parasitic properties should be kept as negligible as possible by proper choice of the time constant. For further reading, reference [38] is recommended.

Besides fully analog and mixed analog-digital realizations of chaotic dynamical systems, there are other possibilities for the experimental verification of the long-term structural stability of a chaotic circuit’s regime. A Field Programmable Analog Array (FPAA) development kit usually offers a graphical user interface and limited-size window where pre-defined building blocks such as ’summators’, filters, and integrators can be easily placed and interconnected. In [39], both analog computer-based and FPAA-based realizations of fractional-order chaotic systems are proposed. This fractionality means that one or several differential equations are of non-integer order, usually between zero and one. Fortunately, such mathematical models can be implemented by an analog computer, using a conventional inverting integrator where the capacitor is replaced with a fractal capacitor (a two-terminal device that behaves similarly to a hybrid between a resistor and capacitor) approximated in the frequency domain. Reference [40] shows another option for documenting the existence of chaos as a solution to some dynamical system. A field programmable gate array (FPGA) platform can be considered a bridge between fully analog realization and purely mathematical analysis. In this work, the authors address the interesting problem of the co-existence of equilibrium points and strange attractors. They discover a dynamical system that generates either a self-excited or hidden chaotic attractor depending on the numerical value of an internal parameter. Even this very special feature does not prevent the design engineer from successive realization. Of course, interesting FPGA-based realizations can be found in other publications, such as [41,42,43,44,45].

It should be pointed out that all of the papers mentioned above contain much more valuable parts than circuit realizations of chaotic systems, namely deep numerical investigations, including bifurcation diagrams, the spectrum of Lyapunov exponents, basins of attraction for individual attractors, etc. Moreover, the provided list of papers dealing with the synthesis of chaotic oscillators is by no means complete. For a further study of the design principles leading to chaos generators, we suggest referring to the comprehensive review work [46]. In addition to the numerous papers cited therein, a vast number of valuable papers can also be found through an internet search.

Mem-elements belong to promising circuit components that contain both nonlinearity and internal inertia. Combined with proper additional circuitry, these are good candidates for the generation of robust chaos. Moreover, mem-elements can be included in standard analog computers and broaden their application potential, as suggested in [47].

Many researchers still focus their attention on the problem of finding new chaotic dynamical systems with specific properties or mathematical descriptions of some real physical phenomena. Since circuitry realization and experimental verification are standard components of such scientific papers [48], the number of novel chaotic circuits will likely increase in the near future. Thus, the importance of a versatile analog development platform is guaranteed.

## 3. Construction of a Hybrid Computer

**Figure 1:**
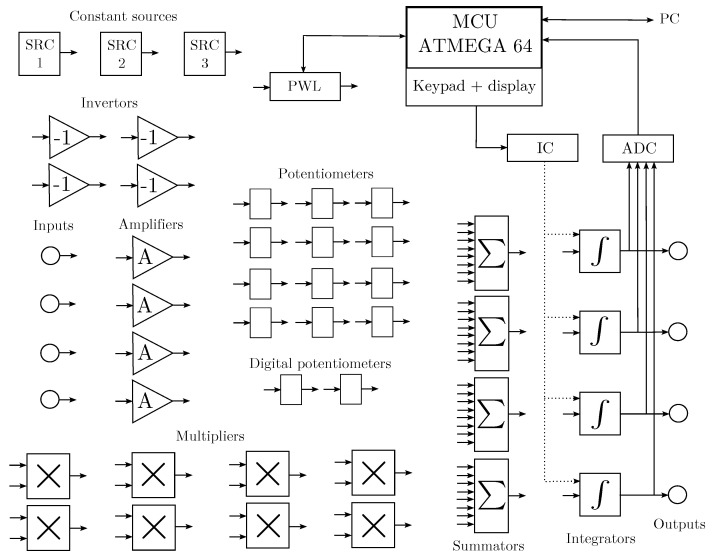
Block diagram of the proposed hybrid computer.

**Figure 2:**
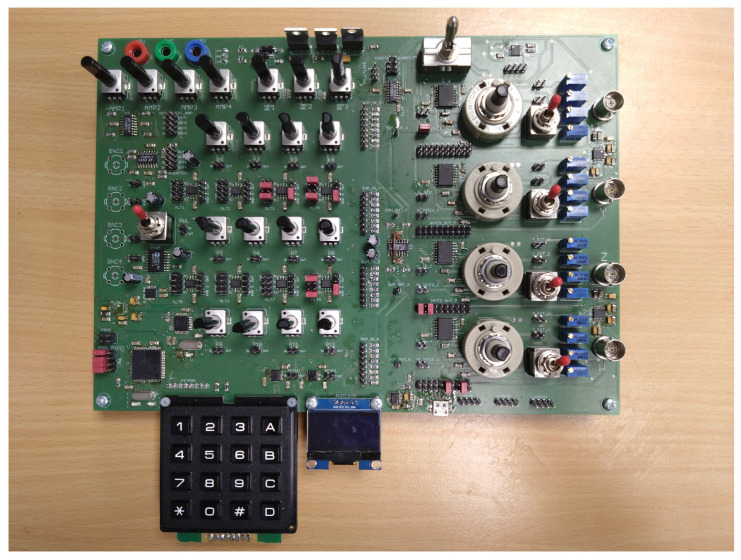
Printed circuit board (PCB) of the proposed hybrid computer.

As briefly mentioned earlier, analog computer techniques can be used to model and simulate nonlinear dynamical systems in the continuous time domain. However, purely analog computers cannot process chaotic signals using digital-based algorithms. In this paper, a hybrid computer is introduced to analyze systems exhibiting chaotic solutions in both the continuous and discrete domains. The hybrid computer is composed of classical analog elements, supplemented by ADCs, digital potentiometers, and control circuits. Figure 1 shows the block diagram of the proposed hybrid computer; Figure 2 shows its practical realization. The individual components of the proposed concept are implemented as separate circuits and then connected by external wires according to the given scheme. The hybrid computer is highly versatile and can be used not only for the simulation of nonlinear dynamic systems. The power supply voltage is symmetrical: ±15 V for the analog part and 5 V for the digital part. Some blocks are designed to be purely analog, while others have analog functions complemented by digital parts, allowing for the setting of individual blocks or the sensing of analog quantities for digital signal processing. Further information, including source files required to rebuild the hybrid computer, can be found at https://www.github.com/mrujzl/Hybrid_computer_v1 (accessed on 27 February 2023) In the following sections, the individual components of the proposed hybrid computer are described in detail.

### 3.1. Integrators and ’Summators’

Integrators are the fundamental analog blocks for solving systems described by ordinary differential equations. The introduced hybrid computer consists of four integrator blocks implemented using commercially available monolithic op-amp AD844. When the compensation terminal is utilized, AD844 acts as a second-generation positive current conveyor (CCII+) with a decoupling voltage follower. The advantage of using AD844 is the ease of implementing a switchable sign of the constant of integration, allowing for both positive and negative integrators to be realized. Another advantage of using this element is the grounded capacitor, which facilitates the realization of initial condition settings without the presence of large leakage currents.

The change in sign is ensured by a lever switch. Next, a simple rotary switch can be used to change the time constant of the integrator.

It is possible to choose from four time constants (1μs, 100μs, 10ms, 1s) and one externally connectable constant. Such a solution allows for the use of an arbitrary value of the time constant or to connect a fractal element to an integration member, increasing the versatility of the computer. The whole integrator can be described as follows:

(1) $$u_{out}\left(t\right)=\pm \frac{1}{RC}\int u_{in}\left(t\right)dt+u_{IC}\left(0\right),$$

where *R* and *C* denote the resistors and capacitors forming the selected time constant, and $u_{IC}$ is the voltage across the capacitors at time t=0 s, i.e., the initial condition of the system, which can be set by the IC block (see below).

The input of individual integrators are usually ’summators’; the number in the presented hybrid computer is the same as the number of integrators, i.e., four summing elements. However, the outputs of these ’summators’ can be disconnected from the inputs of the integrators. This solution is suitable if the simulated system needs to be solved using the method of decreasing the order of derivative (explained in the section below). The summation blocks are implemented by a standard circuit using the LT1209 operational amplifier, which adds the applied voltages with opposite signs. This opposite sign can be compensated by switching integrators. Consequently, the ’summators’ can be described as:

(2) $$u_{out}\left(t\right)=-\sum_{n=1}^{N}u_{n}\left(t\right)$$

where $u_{n}\left(t\right)$ is the individual input voltage. The ’summators’ are designed for eight inputs; therefore, N=8.

### 3.2. Multipliers and Counting-Based Potentiometers

The designed analog computer should be used primarily for simulations of non-linear dynamic systems with chaotic or hyperchaotic behavior. In general, two types of nonlinearities are used in the modeling of these systems, namely piecewise linear (PWL) functions and polynomial nonlinearity. To create polynomial nonlinearities, the analog computer has eight (analog) multipliers that operate in all four quadrants. For this purpose, a well-known integrated circuit AD633 with a supply voltage in a range of ±15 Ṽ is employed. Thanks to a simple dividing constant, it allows for easy implementation of the multiplication of two voltages. The formula of the output voltage is:

(3) $$u_{out}\left(t\right)=\frac{\left[x_{1}\left(t\right)-x_{2}\left(t\right)\right][\left(y_{1}\left(t\right)-y_{2}\left(t\right)\right]}{K}+z\left(t\right)$$

where $x_{1}\left(t\right),x_{2}\left(t\right),y_{1}\left(t\right),y_{2}\left(t\right)$ are the input voltages used to create the final product, z(t) is the input voltage that can be added to the final product, and *K* is the so-called internal division constant (K=10 for the circuit AD633). This constant must be taken into account when programming the analog computer and preparing the programming scheme. However, due to its suitable decimal value, the elimination of this component is easier.

It is not necessary to use analog multipliers only for the realization of nonlinearities. If one of the input voltages is a constant, it is possible to implement a simple multiplication by a constant (e.g., a system parameter) in this way.

The counting-based potentiometers are connected in the form of voltage dividers, which involves a standard connection method used in analog computers. This topology allows for voltage multiplication by a constant in the range of 0 to 1. The solved tasks are typically normalized to the size of the machine unit, which refers to the voltage value that the solution in the state space should not exceed in any dimension. During normalization, the values of the coefficients of the individual terms of the equations are changed, resulting in values typically in the range of 0 to 1. In the designed analog computer, the size of the machine unit is set to 10 V. In addition to standard analog potentiometers, voltage dividers are also implemented using two digital potentiometers, which can be controlled both with internal control elements and with a computer application.

### 3.3. Inverters, Amplifiers, Constant Sources

Some basic circuit blocks that allow for the simulation of dynamic systems in several ways are included in the analog part of the presented hybrid computer. These blocks primarily consist of four inverting operational amplifiers with gain one, which realize an ordinary voltage inverter of the connected variable. In addition, there are four non-inverting amplifiers available, including a potentiometer in the feedback loop. Such a configuration of non-inverting operational amplifiers allows for the realization of multiplication operations by a constant (which can be set in a range of 1 to 5) given by the value of the potentiometer. The use of these blocks makes it possible to normalize the simulated system, even to a variable that does not contain the highest value of the parameter. However, this option requires more experience in the derivation of programming schemes.

Constant voltage sources are the final analog-based components of the proposed concept. They allow for the setting of the output voltage in the range of the analog component’s supply voltage, which is symmetrically ±15 V. In this design, constant voltage sources are utilized for implementing parameters in the analog multipliers (see Section 3.2). Since the multipliers implement formula (3), the output voltage of the constant voltage source can be used as one of the voltages, enabling a wide range of system parameter values to be realized. At the same time, the dividing constants of the multipliers can be exploited for increased versatility.

### 3.4. Digitally Controlled Components

**Figure 3:**
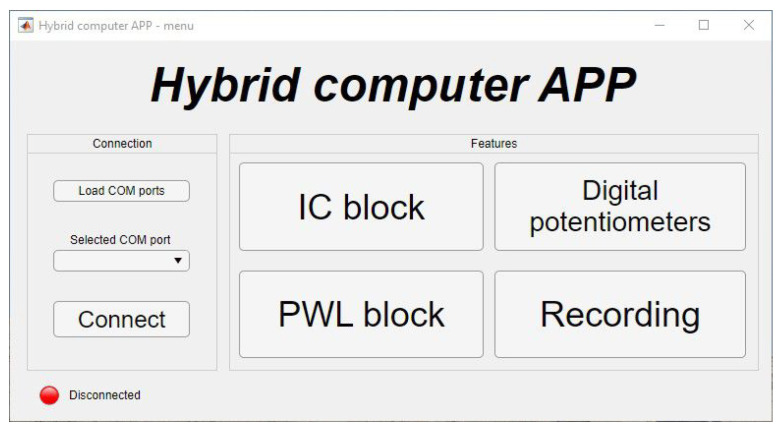
Desktop application for controlling the hybrid computer.

The presented hybrid computer, in addition to the previously mentioned digital potentiometers realizing the so-called counting potentiometers, contains other digital components, such as an IC block, PWL block, and ADCs for monitoring the outputs of integrators (state variables). All digital components are controlled by the main processor ATMEGA64 of the hybrid computer, and can be achieved in two ways. By default, the digital parts can be controlled from an integrated keyboard, while information about the current state of each block is displayed on an integrated OLED display. Another option is to provide control by commands via a personal computer. In order to simplify and improve the work with the command-based system, an appropriate desktop application (graphical user interface), see Figure 3 was created in the interactive development environment *App Designer* (https://www.mathworks.com/products/matlab/app-designer.html) (accessed on 27 February 2023) which is available in the MATLAB program environment.

As mentioned in Section 3.1, the integrator blocks have the ability to disconnect the capacitors and pre-charge them to a specific voltage value, allowing the initial conditions of the system to be set. A digitally controlled initial condition (IC) block is available in the hybrid computer. It is implemented using a digital potentiometer connected as a voltage divider (in the range of the supply voltage) with a decoupling op-amp. In this work, the MAX5437 digital potentiometer from Maxim Integrated is used, which allows its value to be changed in 128 steps. Given a voltage range of 30 V, this corresponds to a change of approximately 0.2 V per step. The IC block is controlled by the main processor via the serial peripheral interface (SPI).

A PWL block is implemented to create nonlinearities using a piecewise-linear function. For this purpose, the commercially available AD844 circuit is connected as a voltage amplifier. By changing the polarity of the amplifier, which is realized by a switch, the quadrants in which the PWL function is located are also changed. The resistor connected to the compensation node of the AD844 circuit is implemented as a digital potentiometer, allowing the gain value to be changed in steps. At the input of the amplifier, the AD7322 analog-to-digital converter is used; with its maximum bipolar range of ±10 Ṽ, it covers the range given by the machine unit.

The whole PWL block is controlled by a separate ATMEGA8 microprocessor unit (MCU). The MCU contains a lookup table (modifiable by a computer application) that includes the voltage sections and their corresponding gain values. If the processor detects a transition between the voltage sections, determined by the lookup table ADC, it changes the resistance of the digital potentiometer to set the gain to the desired value. Controlling this block via the integrated control units would be complicated, so it is the only block controlled via the created desktop application.

Measured results are often required to be in digital form. For this reason, two AD7322 dual-channel ADCs are employed at the outputs of the integrators (state variables, respectively), which allow (as in the PWL block) operating in the 10 Ṽ bipolar range of the machine unit. The ADCs are directly connected to the ATMEGA64 MCU via the SPI bus. The recording function used to collect digital results has two modes. The first mode allows direct control via the integrated keyboard, enabling the control processor to read data from the ADCs with the maximum data rate and send the data directly to the serial line to the computer. This mode sends a raw data stream without any regulation, sending the data regardless of whether they are received or not. In the second mode, connection via the desktop application is assumed. The desktop application, when the *recording mode* option is selected, allows us to set the sampling rate of the ADC from 1 Hz to 50 kHz. At the beginning of the recording, the recorded data are directly displayed in graphs that show all available variable planes (XY, XZ, XW, YZ, YW, ZW). Next, the data (as recorded attractors) can be saved as images (recorded attractors) or as data files (in formats such as CSV, TXT, etc.). Thus, the recorded data can be analyzed offline by other tools or algorithms that are generally used for studying chaotic systems (e.g., Lyapunov exponents or bifurcation analyses).

## 4. Simulations with Hybrid Computers

Various types of calculations and simulations can be performed on analog and hybrid computers. In addition to the already mentioned study of linear and nonlinear dynamical systems, these devices can be used to solve transfer functions, systems of algebraic equations, optimization problems, and find complex roots of polynomials [49]. For this purpose, the following five points must be performed for a successful simulation:

1. Obtaining a mathematical description of the problem to be solved;
2. Decomposition of the mathematical description into a set of operations available on the used device;
3. The transformation of variables involving a limited range of used devices, i.e., the range of a machine unit;
4. Time-variable transformation;
5. Creating and wiring programming schema.

Points 1 and 2 are no different from the problem-solving approaches used in standard computers. Point 3 is usually an essential part of the preprocessing of any system on an analog or hybrid computer. Many problems to be solved have a maximum value size that exceeds the size of the machine unit. Therefore, mathematical equations must be modified so that they do not exceed values that the computer cannot handle. The simplest method for this purpose is the normalization method (already outlined in Section 3.2), which adjusts the values of the parameters of a given system based on the known maximum values that the system can achieve. For instance, the normalization method for a one-dimensional system can be expressed as follows:

(4) $$\left[y\left(t\right)\right]=\frac{y(t)}{N_{y}},$$

where [y] is the variable after normalization and $N_{y}$ is the normalization coefficient, which is usually

(5) $$N_{y}\geq y_{max}.$$

where $y_{max}$ is the maximal value of the state variable.

Point 4 is related to the speed of the solution problem. Some physical problems, such as atmospheric pressure evolution, may take several days to solve realistically. Therefore, it is necessary to adjust the equations to speed up the entire system and obtain a solution within a few minutes. The time transformation of the one-dimensional problem can be expressed as follows:

(6) $$y\left(t\right)=y\left(\frac{\tau }{M_{t}}\right)=Y\left(\tau \right)$$

where τ is the transformed time, Y(τ) is the transformed variable, and $M_{t}$ is the transformation coefficient. However, it is necessary to consider the time transformation from the point of view of mathematical operations, especially in the case of derivatives. The equation for the transformation of derivatives is:

(7) $$\frac{d^{n}Y\left(\tau \right)}{d\tau^{n}}=\frac{1}{M_{t}^{n}}\frac{d^{n}y\left(t\right)}{dt^{n}}.$$

As visible from (7), the coefficients of differential equations in the time transformation can take very different orders of magnitude (due to the exponent *n* of the transformation coefficient). Therefore, in some cases, it is not technically possible to accelerate the system arbitrarily. There should be values in use that can be expressed and set on the device in use.

Point 5 is already related to the creation of wiring; it is advisable to redraw the mathematical expression into a graphical form, which is called a programming schema. Examples of a simple programming scheme are given in the following section about example designs.

### 4.1. Simulation of a Simple Nonlinear Chaotic System

The use of the presented hybrid computer can be illustrated with a simple chaotic system belonging to the so-called JERK system. These are third-order systems that always contain a polynomial nonlinearity, for which analog multipliers on the hybrid computer can be utilized. System ${\mathrm{JD}}_{0}$ [50] was chosen for the simulation, which can be described by the following differential equation:

(8) $$\frac{d^{3}x}{dt^{3}}-a\frac{d^{2}x}{dt^{2}}-b{\left(\frac{dx}{dt}\right)}^{2}-cx=0,$$

where *a*, *b*, and *c* are the parameters of the system and *x* and its derivative (without the third-order derivative) represent the state variables. For further work, it would be useful to rewrite the equation on the operator form using the Laplace operator *p*:

(9) $$p^{3}x-ap^{2}x-px^{2}-x=0.$$

As mentioned in [50], the system generates chaotic behavior at the values of the parameter a=−2.017, b=1, c=−1. Equation (9) can be rewritten with numerical values, as:

(10) $$p^{3}x+2.017p^{2}x-px^{2}+x=0.$$

From the point of view of the used hybrid computer, the advantage of this system is the absence of the steps mentioned in points 3 and 4 (see Section 4), which are usually a part of the preprocessing. The presented hybrid computer has a time constant of 1,s in its integrators and the system is fast enough; hence, it is not necessary to resort to time transformation. Moreover, the maximum values of the state variable in all its derivatives are comparable to the machine unit of 10 V, and therefore normalization is not required. For the presented computer, the appropriate method is to use the method of decreasing the order of the derivative. This method involves the use of integrators connected in a cascade. Feedback with different transfers is used to return the state variables from the outputs of the integrators to the beginning of the cascade, where a “summator” is usually placed and goes to the first integrator of the cascade. To apply the method of decreasing the order of the derivative, it is convenient to adapt the differential equation into a system of differential equations that express the individual state variables:

(11) $$\begin{array}{ccc} p^{2}x & = & \frac{1}{p}\left(-2.017p^{2}x+px^{2}-x\right)\\ px & = & \frac{1}{p}p^{2}x\\ x & = & \frac{1}{p}px. \end{array}$$

**Figure 4:**
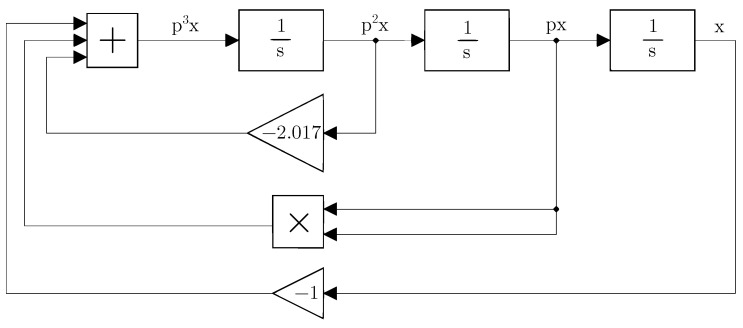
Programming schema according to Equation (11).

The rewritten equation in a system of differential equations clearly shows the cascading order of integrators, the placement of the ’summator’ at the origin of the system, and the feedback connection. Such a modified equation can be represented graphically as a programming scheme (see Figure 4).

Despite the simplicity of the system, it is not necessary to apply points 3 and 4 defined in Section 4. It is necessary to take into account realistic aspects. The presented hybrid computer contains analog multipliers with an internal dividing constant K=10, which is necessary in order to increase the range of input voltages. In addition to the division, the inversion of the result from the ’summator’ must also be taken into account. These aspects must be included in the equation and programming scheme. The inversion of the voltage given by the ’summator’ can be realized by changing the signs of the summed input voltages. In this case, it is even preferable because the linear terms do not need inverters. Similarly, the nonlinear component is not problematic from the viewpoint of the change of sign because the analog multiplier circuit allows implementing the change of sign by an appropriate choice of inputs. The compensation of the division constant is possible by dividing all linear terms by the division constant and then increasing the transfer function (decreasing the time constant in the first integrator after the summator). Such a solution does not change the ratios between the individual parameters, it just reflects the real possibilities of the device used. The Equation (11) can be rewritten as:

(12) $$\begin{array}{ccc} p^{2}x & = & \frac{10}{p}\left(-0.2017p^{2}x+\frac{px^{2}}{10}-0.1x\right)\\ px & = & \frac{1}{p}p^{2}x\\ x & = & \frac{1}{p}px. \end{array}$$

**Figure 5:**
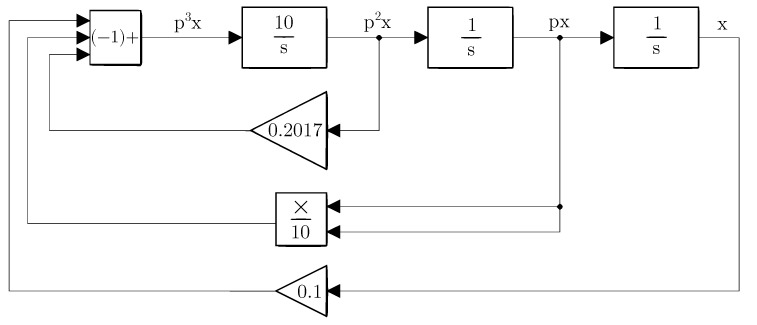
Programming schema with modifications for real use.

**Figure 6:**
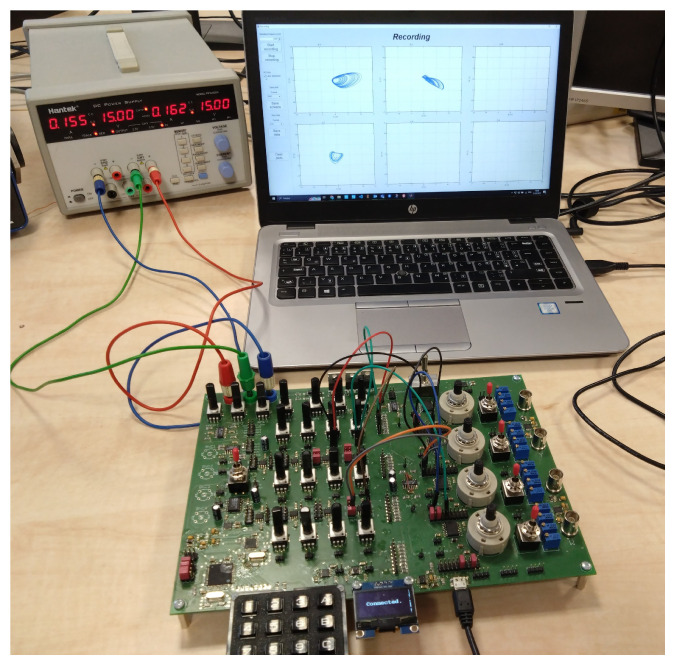
Proposed hybrid computer during measurements of the system ${\mathrm{JD}}_{0}$.

**Figure 7:**
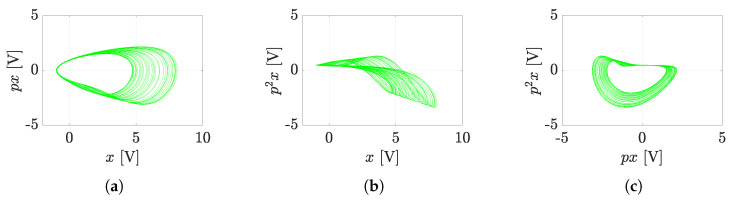
Simulated state attractors of the system ${\mathrm{JD}}_{0}$ (11) by MATLAB; (**a**) [x,px], (**b**) [$x,p^{2}x$], (**c**) [$px,p^{2}x$].

**Figure 8:**
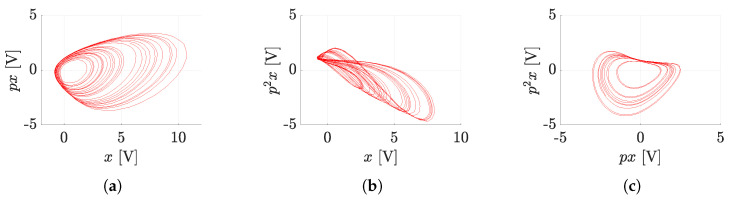
Measured state attractors of the system ${\mathrm{JD}}_{0}$ (12) by the proposed hybrid computer; (**a**) [x,px], (**b**) [$x,p^{2}x$], (**c**) [$px,p^{2}x$].

The modified program scheme (see Figure 5) can already be connected directly to the presented hybrid computer (see Figure 6). The simulation results on the hybrid computer, as well as the results obtained by numerical integration using MATLAB, are the attractors of the state variables, i.e., the attractors of the individual derivative (see Figure 7). The measured waveforms of the attractors in all available projections are shown in Figure 8. They were recorded at a low sampling rate of 10 Hz because it is a slow signal and the programming scheme assumed a low time constant of 1 s. When comparing the measured waveforms with the numerical ones (see Figure 7), only slight differences can be seen, especially in the behavior of the attractor in the [x,px] plane. The observed deformation is mainly due to the low-impedance decoupling found after the computer design. However, despite this shortcoming, the waveforms obtained from the measurement and numerical analysis are very similar, especially in the $\left[px,p^{2}x\right]$ plane. The generated attractor remains structurally stable even when slightly deformed.

**Figure 9:**
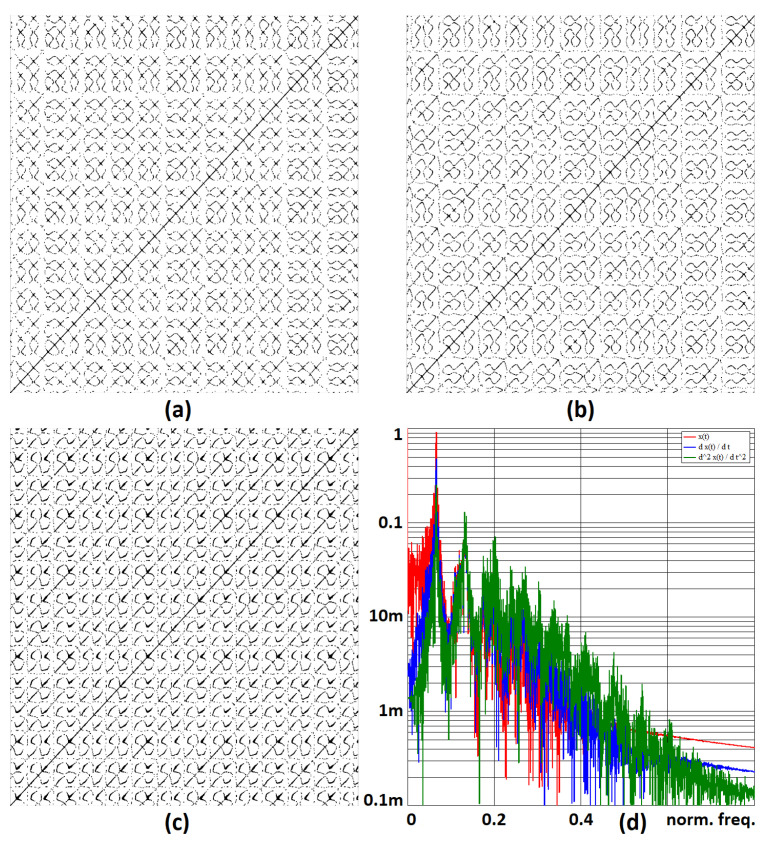
Recurrence plot for the measured chaotic waveform: (**a**) x(t), (**b**) dx(t)/dt, and (**c**) $d^{2}x/dt^{2}$. Subplot (**d**) gives the normalized frequency spectrum of the measured waveforms.

Figure 9 demonstrates recurrence plots for all measured chaotic signals in the sense of the basic definition in [51], i.e., all state variables are considered in the time domain. Both axes of individual plots represent time instances, and a point is plotted if the distance between the compared states is smaller than a value of 0.05. The generated patterns suggest the complex nature of all state variables.

Algebraically simple third-order chaotic systems suitable for testing can be found in [52]. The provided list includes special types of dynamical systems, such as the Sprott case A, which is a conservative system without equilibrium points. The possibility of using an analog computer to generate a chaotic attractor was demonstrated in [53]. The unique property of the chaotic attractor captured by an oscilloscope is its integer Kaplan–Yorke dimension equal to 3. Of course, attractors with lower complexity were also experimentally observed. Moreover, it turns out that there is no need to precisely impose a specific set of initial conditions on the oscillator. The plot showing basins of attraction is very rich in diverse system solutions. Individual areas can be uncovered by the proposed concept of the hybrid analog computer. This could be an interesting topic for future investigations.

### 4.2. Hybrid Analog Computer in Sensor Applications

The hybrid analog computer presented in this paper can be directly used for the synthesis of chaotic circuits based on the Sprott case N, H, and M systems. Therefore, it can serve as a core for a chaos-based sensing system, as described in [54]. Another example of the application of a versatile analog computer is in the construction of a driven chaotic system, which is able to significantly improve the range of a metal detector, as suggested in [55]. The DC signal sensor activated by noise is proposed in [56]. It directly utilizes Chua’s circuit, i.e., the third-order autonomous chaotic circuit with a three-segment piecewise-linear resistor. This system can be easily transformed into a flow-equivalent network based on the integrator block schematic [57]. Moreover, individual parameters of this form of Chua’s oscillator can be tuned by a simple act of the user, and such a change can increase the performance of the sensing system. Versatility (without unnecessary complexity), along with saved construction time, are the main advantages of the proposed hybrid computer.

## 5. Conclusions

This paper thoroughly describes the evolution and construction of a hybrid analog computer, which is a universal tool dedicated to the accurate modeling of nonlinear dynamics up to the fourth order. The developed kit contains both analog and digital parts, with the latter used to build complex transfer functions. The digital block is supplemented by ADC and DAC and also allows the user to impose a specific set of initial conditions into the circuit. Therefore, unlike other realizations of analog computers, our functional example is suitable for modeling nonlinear and chaotic systems, where multiple state attractors can coexist, even for discovering the so-called hidden strange attractors, where the basin of attraction is often narrow and lies far away from the state space origin. Although only a simple chaotic system was experimentally verified, the introduced hybrid analog computer is dedicated to modeling much more complicated dynamical systems, including those with symmetrical vector fields, exhibiting hyperchaotic behaviors, demonstrating trigonometric nonlinearities, etc. Because of its versatility, the developed functional example could be a handy tool for circuit-aided modeling. The hybrid analog computer can be used to realize a complete feedback mechanism of advanced controllers. Suitable interconnection can result in an arbitrary signal generator with adjustable frequency, or significantly improve the overall performance of many sensors on the input analog side [54,55]. From a practical realization point of view, complete guidelines are provided to the readers, including PCB patterns, a list of passive and active circuit components, and design requirements.

## Data Availability

Not applicable.
